# Mechanical Affective Touch Therapy for Anxiety Disorders: Feasibility, Clinical Outcomes, and Electroencephalography Biomarkers From an Open-Label Trial

**DOI:** 10.3389/fpsyt.2022.877574

**Published:** 2022-04-22

**Authors:** Linda L. Carpenter, Eugenia F. Kronenberg, Eric Tirrell, Fatih Kokdere, Quincy M. Beck, Simona Temereanca, Andrew M. Fukuda, Sahithi Garikapati, Sean Hagberg

**Affiliations:** ^1^Neuromodulation Research Facility, TMS Clinic, Butler Hospital, Providence, RI, United States; ^2^Department of Psychiatry and Human Behavior, The Warren Alpert Medical School of Brown University, Providence, RI, United States; ^3^Department of Neuroscience, Brown University, Providence, RI, United States; ^4^Affect Neuro Inc., Brooklyn, NY, United States

**Keywords:** peripheral nerve stimulation, acoustic stimulation, therapeutic neuromodulation, anxiety, EEG

## Abstract

**Background:**

Most external peripheral nerve stimulation devices designed to alter mood states use electrical energy, but mechanical stimulation for activation of somatosensory pathways may be harnessed for potential therapeutic neuromodulation. A novel investigational device for Mechanical Affective Touch Therapy (MATT) was created to stimulate C-tactile fibers through gentle vibrations delivered by piezoelectric actuators on the bilateral mastoid processes.

**Methods:**

22 adults with anxiety disorders and at least moderate anxiety symptom severity enrolled in an open-label pilot trial that involved MATT self-administration using a simple headset at home at least twice per day for 4 weeks. Resting EEG data were acquired before and after a baseline MATT session and again before the final MATT session. Self-report measures of mood and anxiety were collected at baseline, week 2, and week 4, while interoception was assessed pre- and post-treatment.

**Results:**

Anxiety and depressive symptoms improved significantly from baseline to endpoint, and mindfulness was enhanced. EEG metrics confirmed an association between acute MATT stimulation and oscillatory power in alpha and theta bands; symptom changes correlated with changes in some metrics.

**Conclusion:**

Open-label data suggest MATT is a promising non-invasive therapeutic approach to anxiety disorders that warrants further development.

## Introduction

Therapeutic non-invasive peripheral nerve stimulation is being investigated for conditions such as gait disorders (1), pain (2), anxiety, and depression (3, 4). Nerve activation can be achieved by delivering electrical or mechanical energy directly to the area of the dermis that is innervated by the target nerve(s). The majority of potentially therapeutic externally applied devices to date have used electrical stimulation to alter mood states (3–5), address pain (6), and treat diseases (7). Fewer studies have examined the effects of mechanical (acoustic) stimulation; nonetheless, ultrasound (>20 KHz) has been shown to successfully activate peripheral nerves (8, 9) and low-frequency acoustic vibrations (<20 KHz) can activate somatosensory mechanoreceptors. Mechanical stimulation offers a more robust safety profile than electrical stimulation (which itself is considered very low risk) (10, 11), yet somatosensory pathways remain largely unexplored as potential mechanisms for therapeutic neuromodulation.

C-tactile fibers (CT) are specialized unmyelinated Group C peripheral nerve fibers which conduct afferent signals relatively slowly from hairy skin to the insula. CT are mechanoreceptors that show particular sensitivity to gentle touch (12). They fire when stroked at velocities perceived as pleasurable or comforting and prefer temperature ranges that correspond with skin-to-skin interpersonal contact. CT generate signals that mediate emotional rather than discriminative properties of touch (13). This is the target for stimulation by an investigational device called “Mechanical Affective Touch Therapy” (MATT) which delivers gentle vibration on the bilateral mastoid processes.

The acute anxiolytic effect of stimulation observed during device development was presumed to arise from mechanoreceptive signals to the insula, a cortical brain region associated with interoceptive awareness and socio-emotional processing. Insula function and interoception have long been linked to anxiety and mood disorders (14). Interoceptive training has been shown to reduce both somatic symptoms and anxiety states in healthy volunteers (15).

MATT prototype devices use MP3 signal generators wired to a set of digital amplifiers and 3 cm round ceramic piezoelectric actuators which translate the signal to gentle vibrations on the areas of application behind the patient’s ears. Two tractors are mounted on disks and connected by ball joints to a metal headset which also has a cable for attachment to an electronics housing (similar in appearance to a small off-the-shelf MP3 player) enabling the patient to control the amplitude of stimulation. Initial development of the MATT stimulation parameters included various biometric assessments and behavioral questionnaires during tests with different waveforms. Ultimately, an isochronic 10 Hz wave, cycling 2 s on and 2 s off, was chosen for subsequent clinical trials; this stimulation pattern was observed to induce a state of relaxation and increase occipital alpha oscillations in pilot study subjects (data on file Affect Neuro Inc.).

To inform further development of MATT, this open-label pilot study was designed to confirm the preliminary efficacy and feasibility signals in a clinical sample with anxiety disorders and to explore changes in brain activity associated with use. Based on a presumed role of insula in the therapeutic mechanism of action, we assessed interoception (mindfulness) and obtained several electroencephalography (EEG) metrics and resting-state functional magnetic resonance imaging (fMRI) data to elucidate potential MATT mechanisms of action following acute (a single 20-min session) and chronic (4 weeks of twice-daily use) therapy. We hypothesized that chronic therapy would result in reduced anxiety, enhanced mindfulness, and neuroimaging changes that correlated with clinical changes.

Based on preliminary EEG findings that guided the selection of the stimulation parameters during device development, enhanced alpha oscillations were anticipated following acute stimulation. Previous studies have reported power increases in the theta and alpha frequency bands as markers for enhanced mindfulness (16); these metrics were examined in our participants to evaluate the hypothesis that MATT exerts it actions through interoceptive pathways. fMRI data from this study is published separately (17).

## Materials and Methods

### Study Design and Subjects

This was a single site, open-label, 4-week mechanisms-focused study using a prototype device (Affect Neuro Inc.) in outpatients with anxiety disorders recruited through local advertisements. The study was approved by the Butler Hospital Institutional Review Board (IRB) and conducted between 2/13/19 and 10/02/19. Eligible participants were 18–60 years old, determined by a trained clinical rater to meet DSM-5 criteria for an anxiety disorder according to a modified version (updated for DSM-5) of the Mini International Neuropsychiatric Interview (MINI) (18). Eligibility also required a moderate to severe level of current anxiety severity [Generalized Anxiety Disorder 7-item (GAD-7) score ≥ 10] (19). Bipolar I and primary psychotic disorders were exclusionary, as were contraindications to MRI, significant neurological conditions, hospitalization for a psychiatric disorder within the past 6 months, change in psychotropic medication within the past 1 month, and dermatological conditions on the scalp that might be exacerbated by using the device. Eligible participants could be free of psychotropic medications or alternatively remaining on stable regimens; if the latter, they were required to continue the same stable agents and doses for 1 month prior to and throughout the duration of the study. All cases were reviewed by a study psychiatrist for confirmation of eligibility.

### Mechanical Affective Touch Therapy Device and Treatments

**FIGURE 1:**
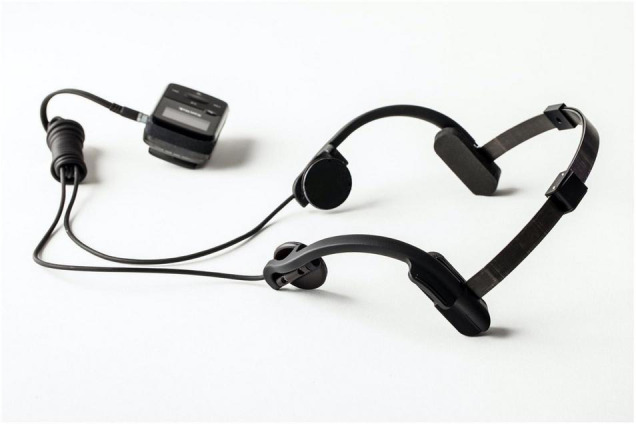
Mechanical Affective Touch Therapy (MATT) prototype device. Stimulating actuators (connected to the MP3 signal generator) are attached to an adjustable metal headset.

The MATT prototype device appears in Figure 1. At baseline, research staff demonstrated how to self-administer the stimulation and helped participants adjust the intensity of the vibrations to a threshold that was consistently detectable but not uncomfortable. Participants were instructed to remain on stable medications/doses throughout the entire study (if applicable) and report any deviations. The first two sessions occurred in the context of baseline biomarker collection procedures and were observed by research staff. Following the second MATT baseline session and demonstration of satisfactory competence in self-administering the treatments, participants were issued a MATT device for home-use and instructed to use it at least twice daily for 20 min at each session. All stimulation was active, and no parties were blinded. Participants were given the option to use MATT for additional sessions each day, if desired. Time of use each day, reason for additional stimulation and/or missed sessions, adverse effects, and technical problems were recorded by participants in daily diaries provided by the study and discussed at an in-person research clinic assessment visit following 2 and 4 weeks of MATT self-administration.

### Assessments

**FIGURE 2:**
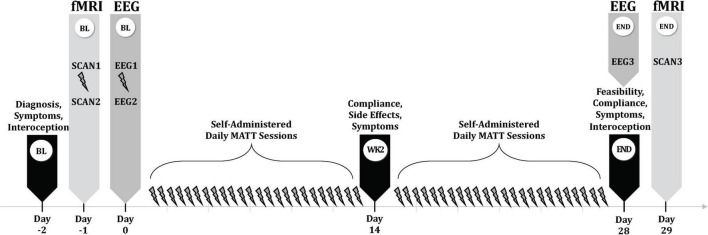
MATT trial assessment timeline. BL, baseline; MATT, Mechanical Affective Touch Therapy; fMRI, functional magnetic resonance imaging; EEG, electroencephalogram; wk, week.

A timeline of study assessments is displayed in Figure 2.

#### Electroencephalography

The first MATT stimulation took place in the MRI research facility, between two scan sessions. Several days later, participants had their second MATT session in the laboratory, with collection of resting EEG immediately before and after a 20-min stimulation session; the vibrating actuators were detached from the headset for the EEG recording session and manually placed on the bilateral mastoid processes beneath the BrainVision neoprene cap which held 32 recording electrodes. For 5 min immediately before (T1 = pre-MATT baseline) and after (T2 = post-MATT baseline) this MATT session, EEG data were acquired with eyes closed in 30 channels. Electrodes Tp9 and Tp10, located in the area of the actuators, were removed to allow for placement of the MATT device during this session. Following 4 weeks of self-administered daily MATT, participants returned to the laboratory for a repeat (endpoint) EEG-MATT session (T3 = pre-MATT endpoint). Acute EEG changes were represented by T2-T1 and chronic changes by T3-T1.

#### Symptoms

Anxiety, depression, and stress symptoms were assessed at baseline, after 2 and 4 weeks of stimulation using several self-report scales: GAD-7, Beck Depression Inventory (BDI) (20), the Perceived Stress Scale (PSS) (21), and the Depression, Anxiety, Stress Scale (DASS) (22).

#### Interoceptive Awareness

The Multidimensional Assessment of Interoceptive Awareness (MAIA) (23) is a 32-item self-report scale developed to measure interoceptive body awareness within eight domains: Noticing, Not-distracting, Not-worrying, Attention Regulation, Emotional Awareness, Self-regulation, Body Listening, and Trusting. MAIA was administered at baseline and endpoint.

#### Feasibility/Side Effects

A paper daily treatment log sheet was given to each participant for recording the time of device use each day, along with any adverse effects or problems. A modified version of the Systematic Assessment for Treatment Emergent Events (SAFTEE) (24) was also used to detect possible side effects at all three assessment visits. A feasibility questionnaire was administered at the final visit. Details of these measures appear in Supplementary Material.

### Data Processing and Statistical Analyses

#### Overall Analytic Plan

The goals of this project included assessment of clinical outcomes associated with MATT and their association to change in biomarkers. We tested change in EEG alpha and theta power associated with an acute (1 session) MATT treatment to confirm signals detected by the device developers in healthy volunteers. Exploratory aims included testing for changes in alpha and theta band metrics following chronic (4 weeks) MATT, and change in EEG markers associated with clinical outcomes. Feasibility, acceptability, and safety of MATT were evaluated to inform future clinical trial designs.

For analysis of symptoms and interoception, participants who were treated with MATT and completed a post-baseline assessment were included in the intent-to-treat (ITT) sample. Last-observation-carried forward (LOCF) values were used for all ITT analyses where week 4 data were missing. Participants who completed week 4 symptom and endpoint EEG assessments comprised the “completer” sample that was used for all EEG analyses.

Statistical tests were two-tailed with an alpha of 0.05. Given the highly exploratory nature of this work, the small sample size, and our goal for detection of potential EEG biomarkers associated with MATT, *p*-values were not adjusted for multiple comparisons on tests of EEG metrics (changes over time or their relation to symptoms). After the application of the Bonferroni factor, significance was defined by *p* < 0.008 on two-tailed tests for measures of mood and anxiety (GAD-7, BDI, PSS, DASS-Depression, DASS-Anxiety, and DASS-Stress). There was only one interoception measure (MAIA total); all *post hoc* tests of individual MAIA subscales (several of which comprised only three items) were considered exploratory and not corrected for multiple comparisons. Results are reported with uncorrected *p*-values below.

#### Analysis of Clinical Outcomes

Features of completers versus drop-outs were compared with independent samples *t*-tests and chi-squares to explore potential baseline differences. Simple descriptive statistics and paired *t*-tests were used to characterize symptom severity at baseline and to evaluate clinical change from baseline to week 4 endpoint (or LOCF) on total scores for each measure (GAD-7, BDI, PSS, and three subscales of the DASS: Depression, Anxiety, and Stress). For each symptom measure, % Change values were calculated for week 4 (or LOCF) data, relative to baseline. Indices of interoceptive awareness (MAIA total, and for *post hoc* tests, 8 MAIA subscale scores) were analyzed similarly.

#### Electroencephalography Metrics

*A priori* EEG metrics of interest were alpha and theta power in frontal and occipital regions. We also explored other markers associated with anxiety: frontal alpha asymmetry (FAA) and individual peak alpha frequency (IAF) (see Supplementary Material). Baseline symptom severity (GAD-7, BDI, PSS, and DASS subscales) and interoception (MAIA) were first examined in association with T1 EEG metrics. Paired *t*-tests were used for EEG changes acutely (T1 vs. T2) and over time (T1 vs. T3). Pearson correlations compared acute (T2-T1) and chronic (T3-T1) change EEG metrics with symptom %Change.

#### Electroencephalography Data Processing

Analyzable EEG data were available from 18 participants at baseline; a subset of 16 had both baseline and endpoint EEG data. EEG data processing methods appear in Supplementary Material.

## Results

### Clinical, Safety, and Feasibility Outcomes

**FIGURE 3:**
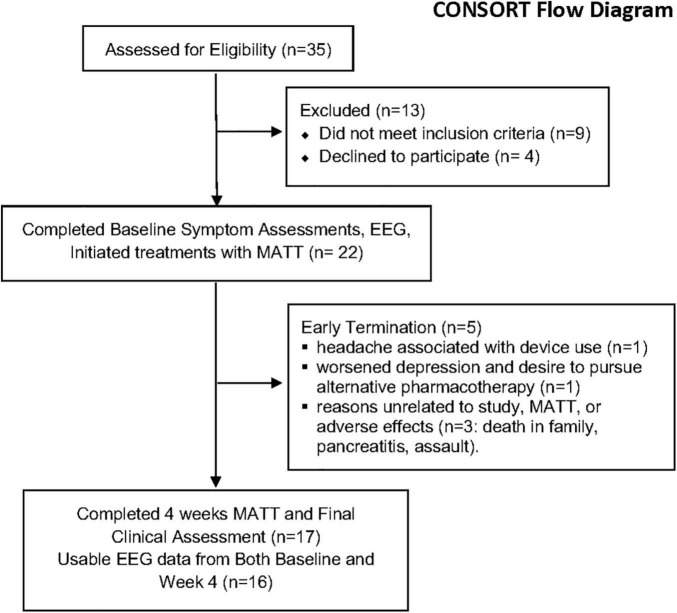
Flow chart demonstrating participant selection and retention. CONSORT, Consolidated Standards of Reporting Trials; MATT, Mechanical Affective Touch Therapy; EEG, electroencephalogram.

A CONSORT flow diagram is presented in Figure 3. Baseline demographic and clinical characteristics for the ITT sample (*n* = 22) appear in Table 1. In the completer sample, mean scores on all symptom measures fell significantly (all *p* < 0.01), and MAIA total increased (*p* = 0.014) (Table 2). Based on diary entries (see Supplementary Material for details), mean MATT compliance with the prescribed dose for ITT ranged from 9 to 100%; among the 17 completers, compliance was 91 ± 13%. There was no significant correlation between compliance estimates and % Change on any symptom measure or any of the EEG metrics we evaluated.

**TABLE 1:** Sample characteristics (*n* = 22).

Age [range; mean (SD)]	18–59; 37.3 (14.8)
**Gender [*n* (%)]**	
Male	5 (22.7%)
Female	16 (72.7%)
Non-binary or trans	1 (4.5%)
**Race [*n* (%)]**	
White	17 (77.3%)
Black	2 (9.1%)
Asian	1 (4.5%)
Other	2 (9.1%)
**Employment status (not mutually exclusive) [*n* (%)]**	
Student	7 (27.3%)
Disabled	3 (13.6%)
Employed full time	10 (45.5%)
Employed part-time	4 (13.6%)
Unemployed	3 (18.2%)
**Current Diagnoses (not mutually exclusive) [*n* (%)]**	
Generalized Anxiety Disorder	21 (95.5%)
Major depressive episode	10 (45.5%)
Panic Disorder	6 (27.3%)
Social Anxiety Disorder, Generalized	11 (50.0%)
Social Anxiety Disorder, Non-generalized	1 (4.5%)
Obsessive Compulsive Disorder	5 (22.7%)
Post-Traumatic Stress Disorder	3 (13.6%)
**Baseline Symptom Severity**	
GAD-7 [mean (SD)]	14.5 (2.2)
Perceived Stress Scale [mean (SD)]	36.2 (5.5)
Beck Depression Inventory [mean (SD)]	30.5 (7.6)
DASS-D Depression Scale [mean (SD)]	20.4 (9.0)
DASS-A Anxiety Scale [mean (SD)]	13.8 (7.9)
DASS-S Stress Scale [mean (SD)]	21.8 (7.9)
**Interoceptive Awareness**	
MAIA Scale Total [mean (SD)]	84.4 (18.0%)
**Medications**	
On stable doses of antidepressants/anxiolytics	16 (72.7%)
Not on any psychiatric medications	6 (27.3%)

**TABLE 2:** Change in symptoms and interoception for completers (*n* = 17).

	Baseline mean (SD)	Week 4 mean (SD)	*t*-value	*p*-value
GAD-7	14.3 (2.2)	7.1 (4.5)	−5.62	0.00003*
Perceived Stress Scale	34.9 (4.5)	26.2 (6.0)	−5.92	0.00002*
Beck Depression Inventory	30.6 (7.7)	14.8 (11.5)	−5.59	0.00003*
DASS-D Depression Scale	19.7 (7.6)	10.1 (8.8)	−4.07	0.00079*
DASS-A Anxiety Scale	13.5 (6.8)	6.4 (4.6)	−3.87	0.0012*
DASS-S Stress Scale	20.6 (7.5)	10.6 (7.7)	−4.30	0.00048*
MAIA Interoceptive Awareness	83.1 (17.3)	93.5 (25.9)	−2.76	0.014

*Uncorrected for multiple comparisons; application of Bonferroni factor results in a corrected threshold of p < 0.008 for statistical significance in measures of mood, anxiety, and stress.

There were no serious adverse events. Treatment-emergent events (at least moderate severity) were heart palpitations (*n* = 2, resolved by week 4), stuffy nose (*n* = 1), headache (*n* = 1; reported at week 2, attributed to device, and associated with study discontinuation), and weakness/fatigue (*n* = 1, reported at week 4). Feedback from participants confirmed feasibility and acceptability. The MATT device was found easy to use, with most of the treatments administered at home when alone. Out of 22 participants, 20 (91%) indicated that they would recommend MATT to others, and 17 (77%) indicated that they would request a prescription for MATT if it received regulatory approval.

### Electroencephalography Markers

#### Alpha Power

At baseline, higher (absolute) T1 frontal alpha power (FAP) correlated with more severe symptoms on the DASS-Anxiety scale (*r* = 0.566; *p* = 0.017) and the DASS-Stress scale (*r* = 0.741; *p* < 0.001; Supplementary Figure 1); it was weakly associated with lower baseline values on the MAIA “Not-Worrying” subscale (*r* = −0.469; *p* = 0.049; Supplementary Figure 1).

**FIGURE 4:**
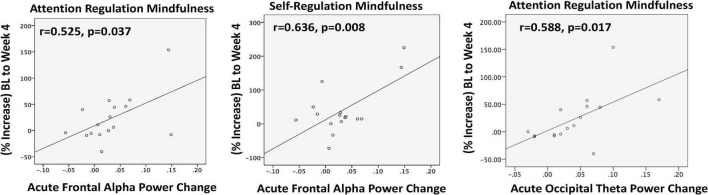
Resting EEG was recorded immediately before and immediately after a 20 min stimulation session at baseline. In completers (*n* = 16), acute increases in frontal alpha power (left and center) and in occipital theta power (right) during the baseline MATT session correlated with enhanced mindfulness after 4 weeks of daily MATT (right). BL, baseline; MATT, Mechanical Affective Touch Therapy; EEG, electroencephalogram.

Acutely, there was a non-significant trend toward increased mean FAP (*t* = 1.89; *p* = 0.076) at T1. A trend suggested larger FAP increases tended to correspond with greater increases in overall mindfulness (MAIA total) at week 4 (*r* = 0.480; *p* = 0.060). *Post hoc* exploration of MAIA subscales showed that the degree of acute FAP increase at T1 correlated with the extent of mindfulness increase after 4 weeks in Attention Regulation (*r* = 0.525; *p* = 0.037) and Self-Regulation (*r* = 0.636; *p* = 0.008) (Figure 4). While mean FAP from baseline to week 4 did not significantly change, greater reductions in perceived stress were associated with dampening of FAP following chronic MATT (*r* = −0.700; *p* = 0.003; Supplementary Figure 1).

**FIGURE 5:**
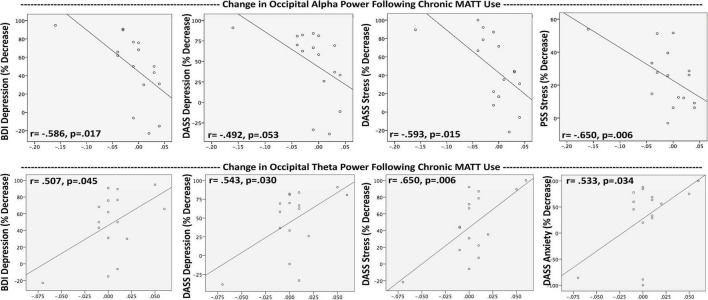
Greater symptom improvement after a 4-week course of MATT was associated with reductions in occipital alpha power and increases in occipital theta power. In completers (*n* = 16) Resting EEG and symptom assessments were collected at baseline and after 4 weeks of daily MATT use. Negative values on the x-axes reflect decreases in resting power from baseline to study endpoint, positive values reflect enhanced power over time. Trend-level relationships are shown here to illustrate the reciprocal direction of change on the same measure for alpha vs. theta power. MATT, Mechanical Affective Touch Therapy; BDI, Beck Depression Inventory; DASS, Depression Anxiety Stress Scale; PSS, Perceived Stress Scale.

Baseline occipital alpha power (OAP) did not correlate with any baseline symptom measures, and the group mean did not change following the baseline stimulation session. Chronic OAP decrease correlated with the degree of symptom improvement on BDI (*r* = −0.586; *p* = 0.017), DASS-Depression (*r* = −0.492; *p* = 0.053), DASS-Stress (*r* = −0.593; *p* = 0.015), and PSS (*r* = −0.650; *p* = 0.006) (Figure 5). OAP decrease over 4 weeks was also found to be linked to the extent of mindfulness increase on the MAIA Noticing scale (*r* = −0.612; *p* = 0.012).

#### Theta Power

At baseline, frontal theta power (FTP) correlated only with perceived stress (PSS; *r* = 0.616, *p* = 0.007). Acute stimulation produced a significant increase in occipital theta power (OTP) (*t* = 3.190, *p* = 0.005), and the degree of change correlated with mindfulness enhanced attention regulation at week 4 (*r* = 0.588; *p* = 0.017; Figure 4). At week 4, the group mean for OTP was generally unchanged, but there were significant correlations between degree of OTP increase and symptom reductions with chronic MATT in depression (BDI *r* = 0.507, *p* = 0.045; DASS-Depression *r* = 0.543, *p* = 0.030), stress (DASS-Stress *r* = 0.650, *p* = 0.006), and anxiety (DASS-Anxiety *r* = 0.533, *p* = 0.034) (Figure 5).

## Discussion

In this report, we describe preliminary clinical outcomes, EEG biomarker data, and feasibility data from a 4-week, open-label pilot trial of a novel non-invasive neuromodulation therapy for individuals with anxiety disorders. We sought to replicate the EEG alpha power changes in association with acute MATT stimulation that were observed in healthy volunteers during device development and guided the selection of parameters for treating anxiety. Mindfulness techniques, which enhance interoceptive awareness, are associated with acute increases in alpha power, reflecting a relaxed state (16, 25, 26). Since interoception is a proposed pathway for MATT’s anxiolytic and mood-improving effects, we expected stimulation would generate related signals in alpha EEG metrics. Generally consistent with early developers’ observations, we found that an initial 20-min stimulation was associated with a non-significant trend toward enhanced FAP in our relatively small sample. There may have been a ceiling effect, as baseline resting alpha power was highly correlated with severity of stress and anxiety symptoms. After 4 weeks of daily MATT use, significant decreases in occipital alpha power (relative to baseline) were found among those who reported the greatest reductions in symptoms.

Increased theta power has also been shown to be a marker of a mindful state during meditation in a number of studies (16, 25). Researchers evaluating neuronal oscillations associated with somatosensory processes for pain versus touch found that, in contrast to EEG response to pain intensity, the intensity of brief touch was encoded only by theta activity (27).

Consistent with a potential mechanism that involves both CT and interoception, we observed increased theta power in the occipital region immediately following a baseline MATT session. Of particular relevance to the therapeutic application considered here for MATT, acute enhancement of theta power, including or prominently in posterior brain regions, has also been shown to occur with transcutaneous trigeminal nerve stimulation (28), transcranial magnetic stimulation (29), and magnetic seizure therapy (30).

Enhanced interoception following 4 weeks of treatment was consistent with the proposed mechanism for MATT’s anxiolytic effect and may implicate activation of insula. We observed increased mindfulness scores over 4 weeks corresponding with decreases in other symptom measures and EEG biomarkers, though some were trend-level findings and *post hoc* exploratory analyses. These preliminary signals define areas for subsequent inquiry and replication. More rigorously designed studies and analyses in larger samples are needed to further evaluate interoceptive processes as a putative therapeutic mechanism.

Though they must be interpreted in the context of an open-label design, the results also confirmed significant improvements in mood, stress, and anxiety symptoms over 4 weeks in a sample of adults with moderate baseline anxiety diagnosed with a range of anxiety disorders. Consistent with many naturalistically treated populations, GAD was prominent, and comorbid major depression characterized nearly half of the sample. The majority were on antidepressant or anxiolytic medications, but a notable portion was free of psychiatric medications and specifically seeking alternatives to pharmacotherapy for symptom management. Participants found use of MATT to be acceptable and were generally compliant with self-administering the 20-min sessions 1–2 times per day in the comfort of their home or other environment of their choice.

We gathered usability and feasibility data to guide further device development. Headache, reported by one subject at the mid-study assessment and associated with discontinuation, was the only notable side effect. Unfortunately, this subject did not report the event to the study team earlier, as the headache associated with MATT would likely have been resolved by turning down the intensity of the stimulation.

Future studies of devices like MATT which are self-administered at home would benefit from concurrent use of smartphone apps or online platforms through which participants can conveniently provide feedback about side effects after each session and receive additional instructions to troubleshoot technical difficulties or address adverse experiences with the device. Overall, the safety data collected in this small study confirmed MATT has a benign safety profile, supporting its further development as a treatment that can be administered with minimal medical monitoring.

A number of limitations characterize this study design and interpretation of the results. This was a single-arm open-label pilot study. As a critical goal of this pilot study was to detect preliminary biomarker signals associated with MATT through neuroimaging that might speak to its proposed therapeutic mechanisms, we employed a generally exploratory analytic approach that focused on magnitude of symptom change over time in relation to corresponding changes in biomarkers. While EEG metrics may be less vulnerable to placebo effects than mood/anxiety symptom change during a clinical trial, it will not be possible to know whether our biomarker observations are attributable to MATT until they are replicated with a sham-controlled design. Given the large number of EEG metrics examined and the lack of *p*-value correction for EEG metrics, it is possible that we detected and reported oscillatory change signals which represent spurious findings. Nevertheless, the findings provide some preliminary support for further target engagement studies examining interoception pathways and anxiety/stress symptoms with MATT. Further supporting this conclusion are MRI data from this study showing that MATT was associated with acute and chronic connectivity increases in insula and posterior regions of the default mode network, respectively, particularly when there were decreases in stress and depression symptoms (17).

The data are also limited in their ability to elucidate relationships between MATT “dose” and other outcomes. We employed a relatively crude method for monitoring MATT dose, i.e., participants were told to self-record their daily use (minutes) of the device in a paper diary and return it at study visits; such data lack the level of accuracy and reliability that will be needed to properly investigate dose-response relationships. Evaluating feasibility was a goal of this study, so we did not compensate participants based on the number of self-administered sessions they logged or otherwise incentivize them to falsify their reports of MATT use. With our simple self-report method and through calculated estimates of each participant’s dose received (relative to the optimal/prescribed dose of 20 min twice daily for 4 weeks), we found compliance was generally good but variable across subjects, as might be expected for an intervention that is self-administered outside of a medical setting multiple times per day. Future studies will benefit from more sophisticated methods for remotely monitoring daily MATT compliance, tracking cumulative dose for individual participants, and capturing if/how use of the device on an “as needed” basis may be acutely anxiolytic.

Confirmation of a C-tactile afferent pathway for insula activation as a putative therapeutic mechanism of MATT would inform its potential applicability in a wide range of disorders where interventions such as meditation and mindfulness have shown promise. Notwithstanding the limitations described here, our results suggest that this specific “affective touch” pathway may provide a way to deliver neurostimulation in a novel manner that appears to have few side effects and does not entail electrical or electromagnetic energy.

## Conclusion

Data from this open-label trial suggest MATT is a promising non-invasive therapeutic approach to anxiety disorders. Acute and chronic use of the investigational MATT device had reciprocal effects on alpha and theta oscillatory activity that corresponded with clinical improvement in this small pilot study. However, double-blind controlled trials and replication samples are needed to definitively establish efficacy and more rigorously replicate proposed therapeutic mechanisms.

## Data Availability Statement

The raw data supporting the conclusions of this article will be made available by the authors, without undue reservation.

## Ethics Statement

The studies involving human participants were reviewed and approved by the Butler Hospital Institutional Review Board (IRB). The patients/participants provided their written informed consent to participate in this study.

## Author Contributions

SH conceived the basic idea. LC and SH designed the study. LC directed the study at Butler Hospital and drafted the manuscript. ET, EK, FK, and AF collected the clinical and EEG data. EK, LC, SG, AF, and ST conducted the data quality control procedures and performed the statistical and data analyses. QB, LC, AF, and EK created the figures and tables. LC, EK, QB, SG, and ST provided input on data analysis and interpretation of results. All authors contributed to the revisions of the manuscript and read and approved the final manuscript.

## Author Disclaimer

The content is solely the responsibility of the authors and does not necessarily represent the official views of the National Institutes of Health.

## Conflict of Interest

Collection of the data for this investigator-designed study was supported by Affect Neuro Inc., developer of MATT therapy, via a contract with Butler Hospital that covered research staff effort, subject payments, and other costs associated with data collection and analysis. In the past 2 years, LC served as a consultant to Neuronetics, Inc., Nexstim PLC, Affect Neuro Inc., Neurolief Ltd., Sage Therapeutics, Otsuka, Sunovion, and Janssen Pharmaceuticals, Inc. and received research support (contracts to Butler Hospital) from Neuronetics, Inc., NeoSync, Inc., Nexstim PLC, Affect Neuro Inc., Neurolief Ltd., and Janssen Pharmaceuticals, Inc. SG is an employee of Affect Neuro Inc. and has a patent No. 17/026,26 pending to Affect Neuro Inc. SH is an employee of Affect Neuro Inc. and has pending patents Nos. 17/026,26 and 16/241,227 to Affect Neuro Inc., and patent 10,786,666 issued to Affect Neuro Inc. The remaining authors declare that the research was conducted in the absence of any commercial or financial relationships that could be construed as a potential conflict of interest.

## Publisher’s Note

All claims expressed in this article are solely those of the authors and do not necessarily represent those of their affiliated organizations, or those of the publisher, the editors and the reviewers. Any product that may be evaluated in this article, or claim that may be made by its manufacturer, is not guaranteed or endorsed by the publisher.
