# Corrigendum: Mechanical affective touch therapy for anxiety disorders: Feasibility, clinical outcomes, and electroencephalography biomarkers from an open-label trial

**DOI:** 10.3389/fpsyt.2022.1010954

**Published:** 2022-09-08

**Authors:** Linda L. Carpenter, Eugenia F. Kronenberg, Eric Tirrell, Fatih Kokdere, Quincy M. Beck, Simona Temereanca, Andrew M. Fukuda, Sahithi Garikapati, Sean Hagberg

**Affiliations:** ^1^Neuromodulation Research Facility, TMS Clinic, Butler Hospital, Providence, RI, United States; ^2^Department of Psychiatry and Human Behavior, The Warren Alpert Medical School of Brown University, Providence, RI, United States; ^3^Department of Neuroscience, Brown University, Providence, RI, United States; ^4^Affect Neuro Inc., Brooklyn, NY, United States

**Keywords:** peripheral nerve stimulation, acoustic stimulation, therapeutic neuromodulation, anxiety, EEG

In the published article, there was an error. We incorrectly stated that the piezoelectric actuators were 30cm in diameter when they were 3cm in diameter.

A correction has been made to **Introduction**, Paragraph four. This sentence previously stated:

“Mechanical Affective Touch Therapy prototype devices use MP3 signal generators wired to a set of digital amplifiers and 30 cm round ceramic piezoelectric actuators which translate the signal to gentle vibrations on the areas of application behind the patient's ears.”

The corrected sentence appears below:

“MATT prototype devices use MP3 signal generators wired to a set of digital amplifiers and 3 cm round ceramic piezoelectric actuators which translate the signal to gentle vibrations on the areas of application behind the patient's ears.”

The authors apologize for this error and state that this does not change the scientific conclusions of the article in any way. The original article has been updated.

## Publisher's note

All claims expressed in this article are solely those of the authors and do not necessarily represent those of their affiliated organizations, or those of the publisher, the editors and the reviewers. Any product that may be evaluated in this article, or claim that may be made by its manufacturer, is not guaranteed or endorsed by the publisher.

