# Supplementary material for: Mechanical Affective Touch Therapy for Anxiety Disorders: Feasibility, Clinical Outcomes, and Electroencephalography Biomarkers From an Open-Label Trial
Source: Front Psychiatry. 2022 Apr 22;13:877574. doi: 10.3389/fpsyt.2022.877574 (PMC9072623; doi:10.3389/fpsyt.2022.877574)
Supplement: Supplementary file 1 [file Data_Sheet_1.docx]

Mechanical Affective Touch Therapy (MATT) for Anxiety Disorders: Feasibility, Clinical Outcomes, and EEG Biomarkers from an Open-Label Trial

**Supplemental Material**

**Assessment of Side Effects, Feasibility, and Compliance**

Daily treatment log sheets completed by participants were reviewed by research staff at the week 2 and week 4 visits so additional detail could be solicited when appropriate. A modified version of the Systematic Assessment for Treatment Emergent Events (SAFTEE) scale (1) detected possible side effects at all three assessment visits. The list of 55 side effects commonly associated with psychiatric treatments was presented as a self-report measure, and participants rated the impact of each item on a 0 (none) to 3 (severe) scale. Any item with an increase in severity rating to “moderate” or “severe” at week 2 or week 4 was considered a treatment-emergent event.

A feasibility questionnaire was customized for this particular device, with fixed response options and also open text fields. The feasibility questionnaire was administered as a self-report measure at the final visit to obtain participants’ feedback about their general experience and comfort level with the MATT device, reasons for missed sessions, reasons for adjusting the intensity of stimulation (if applicable), characteristics of the environment when/where MATT was typically used, etc. A compliance rating was calculated for each participant (ranging from 0 to 100%) based on the prescribed dose of MATT (two sessions per day x 28 days) and the number of recorded self-administered sessions reported in the log.

**Participant Disposition**

A total of 35 participants consented and were screened; 9 did not meet eligibility criteria, and 4 declined to participate. A total of 22 participants completed baseline symptom assessments, EEG data collection, and were treated with MATT; however, technological problems resulted in unusable EEG data for one subject. Seventeen of the 22 who initiated the course of MATT completed the week2 and final week4 assessments. Five participants terminated the study early: one due to headaches associated with the device, one due to worsening depression symptoms and desire to pursue an alternative pharmacotherapy; the other 3 discontinued for reasons unrelated to the study treatment or side effects (death in the family, pancreatitis, and victim of assault). There were no differences between the 17 completers and 5 drop-outs on any baseline demographic or symptom severity measures.

Daily use ranged from 0-3 MATT treatments per day (mean±SD 1.7±0.4). Total number of treatment sessions across the 4-week course (or until early termination) ranged from 5 to 67 (mean±SD 49.1±17.1 for ITT; 51.1±14.8 for completers).

**EEG Data Preprocessing**

EEG data was processed in EEGlab, a MATLAB extension (2, 3). Raw data were examined for bad channels and the first and last 10-20 seconds of the EEG recording were rejected to account for time when a research assistant was entering and exiting the recording room. Channels Tp9 and Tp10 (and any other bad channels) were removed for all analyses because the stimulating actuators were in those positions.

In preparation for absolute power analyses, after visualizing data with EEGLAB for artifact removal, signals were preprocessed with Butterworth filters (0.5 Hz – 35 Hz, order-10). EEG blocks for each individual channel were divided into equal length segments, and fast fourier transform (fft, MATLAB) was applied to each segment. The absolute magnitude of power in the alpha frequency band (7.5-12.5Hz) and theta frequency band (3.5-7.5Hz) was calculated using a trapezoidal integration function (trapz, MATLAB).

For frontal alpha asymmetry and peak individual alpha frequency calculations, Fp1 and Fp2 were excluded and all EEGs were filtered with a band pass filter between 0.01 Hz and 30 Hz. Baseline was removed and data were re-referenced to the average reference. After running ICA, IClabel (an EEGlab extension) was used to not only plot the power spectrum and spatial distribution of all components, but also to label components with their most likely source. This extension uses a neural network trained on thousands of EEG recordings to identify whether a component’s signal is coming from the brain, eye, muscle, heartbeat, or noise (4). Eye movement components were removed if identified. One-second epochs were added to each EEG, and any epochs with artifact were rejected manually. Each 5-minute recording had at least 160 epochs to ensure reliability for frontal alpha asymmetry (5). Using the spectopo function and 2s Hanning windows that overlap by 50%, the power-frequency spectrum for the alpha (7.5 – 12.5 Hz) band was calculated for each window.

**Frontal and Occipital Power**

Due to markedly higher impedances in the baseline EEG data, two cases were excluded from baseline power analyses. The change in absolute power between T1 and T2 blocks was calculated, and the channels were grouped by lobes for statistical analysis (N=18). Frontal region power was averaged over electrodes Fp1, Fp2, F3, F4, F7, and F8, and occipital region power was averaged over electrodes O1, Oz, and O2. Acute changes (from T1 to T2) are reported in absolute power, but the impedances across electrodes during the baseline EEGs were different from impedances during the endpoint EEGs, so relative power was calculated in order to reliably compare EEG recordings at T1 and T3. Within alpha and theta frequency bands, relative power was calculated by dividing absolute power by total power at each electrode.

**Frontal Alpha Asymmetry (FAA)**

Prefrontal electrodes F3, F4 and F7, F8 were stable for all subjects and therefore used for FAA and IAF analyses, consistent with other studies in the field (6-9). Calculations for FAA and IAF both accounted for differences in impedances, so absolute alpha power could be used for analysis of these two metrics.

A normalized FAA score was calculated for each 2s window such that frontal alpha asymmetry is equal to right hemisphere alpha power minus left hemisphere alpha power divided by total alpha power (6):

$$A = (P_{ɑR}-P_{ɑL})/({P_{ɑR}+P_{ɑL})}$$

where A = alpha asymmetry score; P_ɑR_ = ∑ right alpha power (electrodes F4, F8); and P_ɑL_ = ∑ left alpha power (electrodes F3, F7). FAA scores were averaged across all windows to determine a single FAA score for each EEG recording period.

**Peak Individual Alpha Frequency (IAF)**

The spectral frequency data for each 2s epoch were identified with the spectopo function and averaged across 4 frontal electrodes (F3, F4, F7, F8). A gaussian curve was fitted to the alpha band (7.5-12.5Hz) using the fit function in MATLAB’s Curve-Fitting Toolbox. If an error was found while fitting a curve to any of the epochs, that epoch was assumed to lack significant modulation in the alpha range and excluded (10). For each epoch, the absolute peak of the spectral-frequency curve and the Gaussian-fitted peak were identified and averaged across all epochs. Two frontal IAF values (absolute frontal IAF and Gaussian-fitted frontal IAF) were thus generated for each participant. Because the two values were highly correlated with one another, we report results from only the Gaussian-fitted IAF.

**Mindfulness Clinical Outcomes**

Mindfulness tended to increase over time as reflected by increased MAIA total scores (p=0.014; completer sample); Post-hoc examination of the subscales revealed this finding was attributable to four domains: ‘Not Worrying’ (p=0.026), ‘Self-Regulation’ (p=0.026), ‘Body Listening’ (p=0.013), and ‘Trusting’ (p=0.003). Greater baseline-to-endpoint increases in mindfulness (MAIA total score) during the trial correlated with greater reductions in stress (PSS r=0.46, p=0.044; DASS-Stress r=0.55, p=0.022) and depression symptoms (BDI r=0.63, p=0.007; DASS-Depression r=0.57, p=0.012), confirming a role for increasing mindfulness as a mechanism or correlate of clinical improvement in individuals with anxiety and mood disorders.

**EEG: Frontal Alpha Asymmetry (FAA)**

Similar to previous reports linking right-sided alpha power dominance to anxiety disorders (11), we found that higher alpha power in the right hemisphere relative to the left hemisphere was strongly correlated with symptom severity at baseline. FAA showed significant positive relationships with baseline symptom severity (BDI [r=0.481, p=0.032], DASS Depression [r=0.568, p=0.009], and DASS Stress [r=0.535, p=0.015]) i.e., relatively greater right-hemispheric alpha power was seen in participants who more severely symptomatic. None of the FAA measures (baseline FAA, acute FAA change with one MATT session, nor FAA change following chronic MATT) was significantly related to clinical outcomes.

**EEG: Individual (Peak) Alpha Frequency (IAF)**

Baseline frontal IAF values (mean±SD, 10.15±0.8 Hz) were not significantly associated with symptom severity or mindfulness. None of the IAF measures (baseline IAF, acute IAF change with one MATT session, nor IAF change following chronic MATT) was significantly related to clinical outcomes.

**Feasibility and Safety Outcomes (Additional Detail)**

There were no serious adverse events. Adverse events reported at a moderate or severe level were: weakness/fatigue (n=7), headache (n=2), inability to sit still (n=2), rapid or pounding heartbeat (n=2), muscle cramps or stiffness (n=2), stuffy nose (n=2), and dizziness (n=1). Of these events, at least a mild level of the symptom was already present at pre-treatment baseline in 7 cases; new onset events were limited to heart palpitations (n=2), stuffy nose (n=1), headache (n=1), and weakness/fatigue (n=1).

Our brief feasibility questionnaire was completed by all 22 participants in the ITT sample upon study exit. When asked to rate how difficult it was to use the device on a scale from 0 (“no effort”) to 10 (“impossible”), mean±SD rating was 2.30±2.78. The intensity of stimulation was changed from the initial threshold on one or more occasions by 10 (46%) participants; increases were made by 8 participants to accommodate their activity level or noise in the surrounding environment, while 2 participants found the initial thresholding setting to be too uncomfortable or associated with headache and subsequently turned it down. Half of the participants (n=11) reported feeling comfortable wearing the headset in public or around friends, though 82% (n=18) endorsed a preference to administer MATT when alone. Only 37% (n=13) used the device exclusively in their homes; the remaining also used MATT while in their automobiles or workplaces. All but 2 participants (90.9%; n=20) indicated they would recommend MATT to their friends and a significant majority (77%; n=17) indicated that they would request a prescription for MATT if it were FDA approved.

**References**

1. Levine J, Schooler NR. SAFTEE: a technique for the systematic assessment of side effects in clinical trials. Psychopharmacol Bull. 1986;22(2):343-81.

2. Delorme A, Makeig S. EEGLAB: an open source toolbox for analysis of single-trial EEG dynamics including independent component analysis. J Neurosci Methods. 2004;134(1):9-21.

3. Matlab Release 2010a. Natick, Massachusetts, United States: The MathWorks Inc.

4. Pion-Tonachini L, Kreutz-Delgado K, Makeig S. ICLabel: An automated electroencephalographic independent component classifier, dataset, and website. Neuroimage. 2019;198:181-97.

5. Towers DN, Allen JJ. A better estimate of the internal consistency reliability of frontal EEG asymmetry scores. Psychophysiology. 2009;46(1):132-42.

6. Allen JJ, Coan JA, Nazarian M. Issues and assumptions on the road from raw signals to metrics of frontal EEG asymmetry in emotion. Biol Psychol. 2004;67(1-2):183-218.

7. Blackhart GC, Minnix JA, Kline JP. Can EEG asymmetry patterns predict future development of anxiety and depression? A preliminary study. Biol Psychol. 2006;72(1):46-50.

8. Coan JA, Allen JJ. Frontal EEG asymmetry as a moderator and mediator of emotion. Biol Psychol. 2004;67(1-2):7-49.

9. Harrewijn A, Van der Molen MJ, Westenberg PM. Putative EEG measures of social anxiety: Comparing frontal alpha asymmetry and delta-beta cross-frequency correlation. Cogn Affect Behav Neurosci. 2016;16(6):1086-98.

10. Haegens S, Cousijn H, Wallis G, Harrison PJ, Nobre AC. Inter- and intra-individual variability in alpha peak frequency. Neuroimage. 2014;92:46-55.

11. Demerdzieva A, Pop-Jordanova N. Relation Between Frontal Alpha Asymmetry and Anxiety in Young Patients with Generalized Anxiety Disorder. Pril (Makedon Akad Nauk Umet Odd Med Nauki). 2015;36(2):157-77.

*
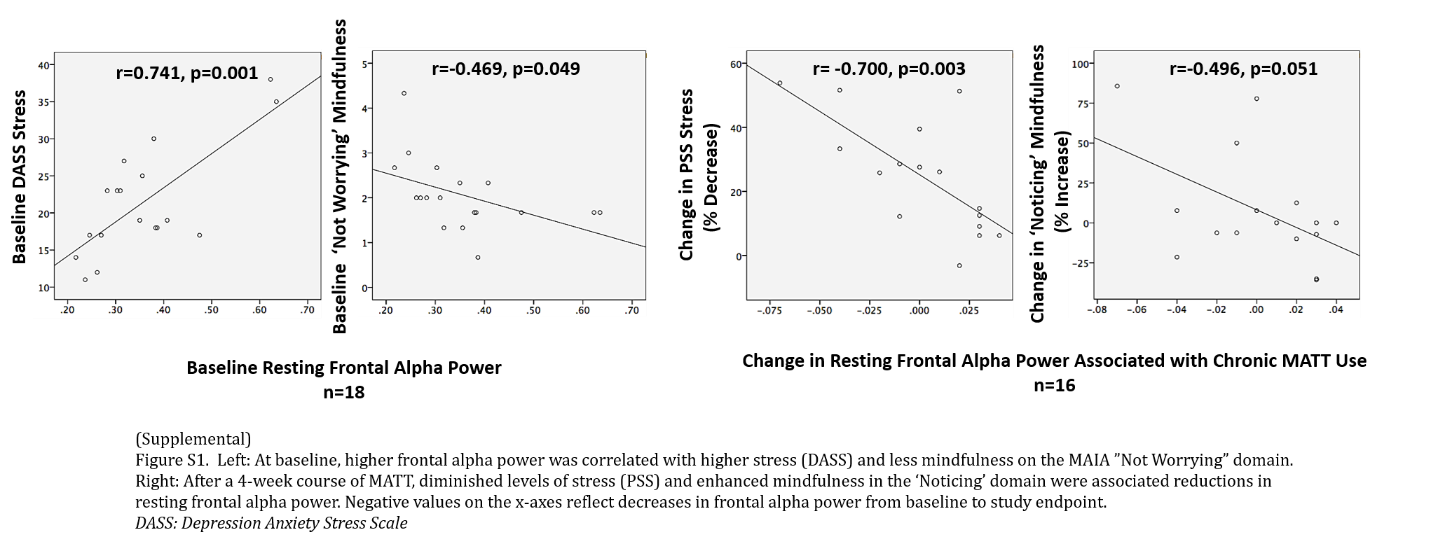
*
